# DAB-quant: An open-source digital system for quantifying immunohistochemical staining with 3,3′-diaminobenzidine (DAB)

**DOI:** 10.1371/journal.pone.0271593

**Published:** 2022-07-20

**Authors:** Sneh Patel, Sara Fridovich-Keil, Shauna A. Rasmussen, Judith L. Fridovich-Keil

**Affiliations:** 1 Emory College, Emory University, Atlanta, GA, United States of America; 2 Department of Electrical Engineering and Computer Sciences, University of California at Berkeley, Berkeley, CA, United States of America; 3 Department of Human Genetics, School of Medicine, Emory University, Atlanta, GA, United States of America; Technion Israel Institute of Technology, ISRAEL

## Abstract

Here, we describe DAB-quant, a novel, open-source program designed to facilitate objective quantitation of immunohistochemical (IHC) signal in large numbers of tissue slides stained with 3,3′-diaminobenzidine (DAB). Scanned slides are arranged into separate folders for negative controls and test slides, respectively. Otsu’s method is applied to the negative control slides to define a threshold distinguishing tissue from empty space, and all pixels deemed tissue are scored for normalized red minus blue (NRMB) color intensity. Next, a user-defined tolerance for error is applied to the negative control slides to set a NRMB threshold distinguishing stained from unstained tissue and this threshold is applied to calculate the fraction of stained tissue pixels on each test slide. Results are recorded in a spreadsheet and pseudocolor images are presented to document how each pixel was categorized. Slides can be analyzed in full, or sampled using small boxes scattered randomly and automatically across the tissue area. Quantitation of sampling boxes enables faster processing, reveals the degree of heterogeneity of signal, and enables exclusion of problem areas on a slide, if needed. This system should prove useful for a broad range of applications. The code, usage instructions, and sample data are freely and publicly available on GitHub (https://github.com/sarafridov/DAB-quant) and at protocols.io (dx.doi.org/10.17504/protocols.io.dm6gpb578lzp/v1).

## Introduction

Quantifying immunohistochemical (IHC) staining with 3,3′-diaminobenzidine (DAB) in large numbers of scanned slides can be a daunting task. Numerous digital platforms are currently available (e.g., Fiji [1], Icy [2], and CellProfiler [3]) that offer a range of functionalities and limitations. One of the first applications, ImageJ (reviewed in [4]), enables sensitive quantitation but requires substantial user input, and therefore possible user bias. QuPath [5], which uses ImageJ and OpenSlide [6] as dependencies, offers numerous features and an excellent user interface, but also requires substantial user input for each slide and sets thresholds for staining based on color peaks in the test slides themselves rather than true negative control slides.

Here we describe a novel, open-source program designed to simplify and automate the process of quantifying DAB staining in bulk numbers of slides organized into folders, yielding largely unbiased results with a minimum of user effort. Our code is written in Python and shared free-of-charge on GitHub (https://github.com/sarafridov/DAB-quant) and at protocols.io (dx.doi.org/10.17504/protocols.io.dm6gpb578lzp/v1).

In this study, we used scans of slides representing liver from GALT-null rats administered an scAAV9-HA.hGALT vector (test), or vehicle alone (negative control), stained with hematoxylin (blue color) and an antibody directed against the transgene HA tag (brown color). By analyzing scans of 6 negative control and 4 test slides we demonstrate functionality, sensitivity, and reproducibility of our DAB-quant system. Of note this software has already been used to generate data published in a peer-reviewed paper [7]. Our Python code should be immediately useful for those wishing to quantify the degree of DAB staining in tissue slides, even slides with imperfections, and because it is open source, our code can be customized by those wishing to adapt it for other applications.

## Materials and methods

### Ethics statement

The rat tissue slides utilized in this study were generated with approval of the Emory University Institutional Animal Care and Use Committee (IACUC) under protocol PROTO201700095 (PI: JL Fridovich-Keil). IRB approval was not required as this study did not involve any human subjects.

### Protocols.io

The protocol described in this peer-reviewed article is published on protocols.io [dx.doi.org/10.17504/protocols.io.dm6gpb578lzp/v1] and a short descriptive text with the relevant links is included for printing as S1 File with this article.

#### Preparation of slides used here

The test and negative control slides used here were prepared as described in Daenzer et al [7]. Specifically, test slides included 5 micron sections of fixed liver tissue derived from GALT-null rats that had been administered an scAAV9-HA.hGALT vector either 14 or 30 days prior to euthanasia. Negative control slides included 5-micron sections of fixed liver tissue from GALT-null rats that had been administered vehicle alone (phosphate-buffered saline, PBS). The individual rats whose samples are presented here include: Rat A (FKRC311.6), Rat B (FKRC297.2), Rat C (FKRC297.12), Rat D (FKRC297.10) and Rat E (FKRC297.9).

Immunohistochemistry (IHC) was performed using an anti-HA monoclonal antibody (Cell Signaling Technology #3724) as primary at 1:1000 dilution, and a peroxidase-coupled goat anti-rabbit IgG (H+L) (Vector Labs PI-1000-1) as secondary. Antibody binding was visualized as brown color by reaction of the coupled peroxidase with 3,3′-diaminobenzidine (DAB; Vector Labs, SK-4100), as recommended by the manufacturer. All slides were also treated with hematoxylin which nonspecifically stained all cells a light blue color. Mounted slides were scanned on a Hamamatsu Nanozoomer 2.0 HT whole slide scanner at 40X and viewed using the free Hamamatsu software (NDP viewer).

### Downloading and using DAB-quant

DAB-quant will run on both Mac and PC platforms. Detailed instructions, downloadable Python code, license information, and sample negative control and test slide scans are freely and publicly available at: https://github.com/sarafridov/DAB-quant. Example run times on both Mac and PC platforms are presented in Table 1.

**Table 1:** Example run times on a PC versus a Mac.

Computer model	Time per control slide	Time per test slide (20 regions)	Time per test slide (full scan)
Dell PC 16.0 GB RAM Intel i7 processor Windows 10	6.1 min	7.8 sec	2.5 min
2020 MacBook Pro 8.0 GB RAM Apple M1 chip macOS Big Sur	3.7 min	7.4 sec	1.5 min

In brief, once all software has been installed, scans of negative control slides are placed in a designated control folder where they are pre-processed and then analyzed to define thresholds for distinguishing empty space from tissue, and DAB-stained from unstained tissue pixels. Scans of corresponding test slides are placed in a designated test folder, and depending on user input, each is either analyzed in full or randomly sampled, with or without exclusion of user-defined problem areas.

In the course of processing, output folders for each test slide are created that contain the following files, some automatic and others optional: (a) a low-resolution image of the entire slide, overlaid with ID-coded sampling boxes, if any, (b) a 2-column text table where the first column lists the sampling box ID code (0 if the full slide was analyzed) and the second column lists the fraction of tissue pixels in that box with a Normalized Red Minus Blue (NRMB) value above the threshold set for defining a pixel as DAB-stained, (c) a histogram showing the proportion of tissue pixels scoring within narrow ranges of NRMB value, combined across the entire analyzed region of the slide, (d) a text file listing the data plotted in the histogram in (c), (e) a text file listing the proportion of tissue pixels by NRMB range for the full slide and for each analyzed region, e.g., sampling box, (f) a "true-color" image of each sampling region, (g) a pseudocolor image of each region in which empty space is shaded green, DAB-stained tissue pixels are shaded pink, and DAB-unstained tissue pixels are shaded gray, and finally (h) a raw histogram for each sampled region showing the proportion of tissue pixels from that region scoring within narrow ranges of NRMB value.

## Results

### Strategy and thresholds

**Fig 1:**
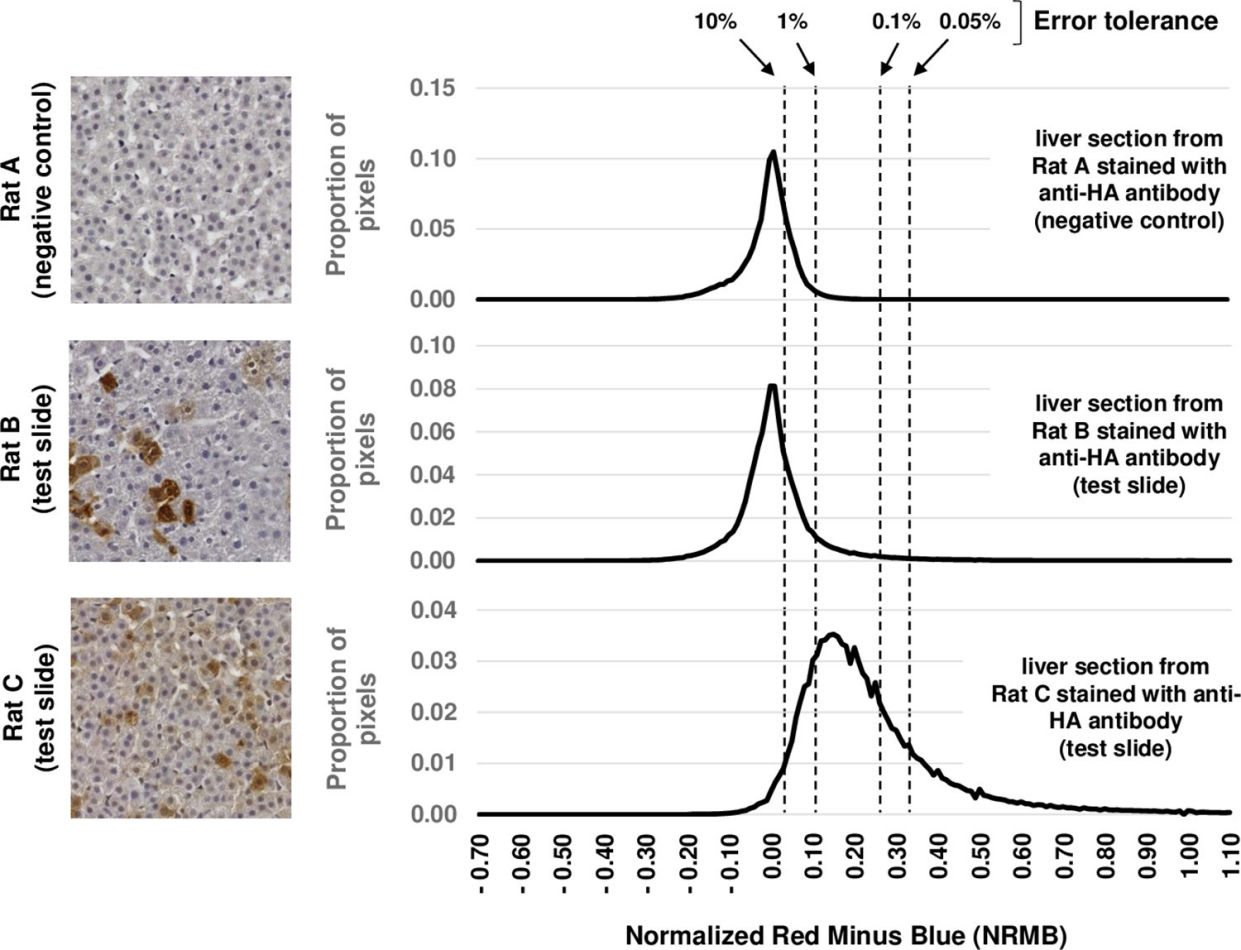
How DAB-quant sets a threshold to distinguish DAB-stained from unstained pixels based on a user-defined error tolerance for pixel misclassification in negative control slides. The true-color images to the left depict representative sampling boxes; histograms to the right present combined pixel data from all 50 sampled regions on the corresponding slide. The NRMB threshold corresponding to each error tolerance limit illustrated was calculated using combined data from all 6 negative control slides. The top row presents data from a single negative control slide. The middle and bottom rows present data from individual test slides.

Each pixel in a color scan holds intensity values between 0 and 255 for each of 3 colors: red, green, and blue. To detect brown DAB staining in tissue sections also stained with hematoxylin (blue), we calculated a normalized red minus blue (NRMB) value for each pixel as follows: $\frac{red-blue}{\max\;\left(1,blue\right)}$. For a pixel that is intensely red, with minimal blue color, the NRMB will be close to 255. For a pixel that has some blue, but no red color, the NRMB value will be -1. In our example test slides, the NRMB values for tissue pixels almost always fell between -1 and +3. Pixels that appeared slightly brown to the naked eye had NRMB values close to 0.5, whereas unstained tissue pixels had an NRMB value of close to -0.1 (Fig 1).

To quantify the fraction of stained pixels in a scanned slide requires the definition of two thresholds: one distinguishing tissue from empty space to be excluded, and the second distinguishing brown-stained tissue from unstained tissue. Here, we define each of these thresholds using 6 negative control slides prepared in parallel with the test slides (see Materials and Methods). To be clear, our negative control slides were stained in parallel with our test slides using exactly the same antibodies and DAB reagents. They were negative controls because the tissues derived from GALT-null rats that had been treated with PBS rather than AAV9 encoding human GALT. The primary antibody therefore had no target to bind, so any brown color was artifactual. If no negative control slides are available, reasonable default thresholds are provided in the code that can be either accepted or adjusted by the user.

If negative control slides are provided, DAB-quant applies Otsu’s method [8], as implemented in scikit-image [9], to define a cutoff distinguishing tissue from empty space. In short, a pixel is classified as empty if it appears white, meaning the average of the 3 color intensities (red, green, and blue) is above a high threshold effectively requiring that all 3 be close to the maximum value of 255. Otsu’s method selects this threshold value to divide a histogram of control pixel intensities into two classes such that the within-class variance is minimized, and the between-class variance is maximized.

To define a threshold for DAB “stain-positive” pixels, our system applies a user-defined error tolerance limit to calculate the NRMB threshold that would count no more than that percentage of tissue pixels in the negative control slides as stained. For example, an error tolerance limit of 0.1% (the default) would erroneously count approximately one pixel out of every 1000 obligate unstained tissue pixels as stained. Setting an acceptable threshold and maintaining it across all slides in a given experiment lends objectivity to the application.

**Fig 2:**
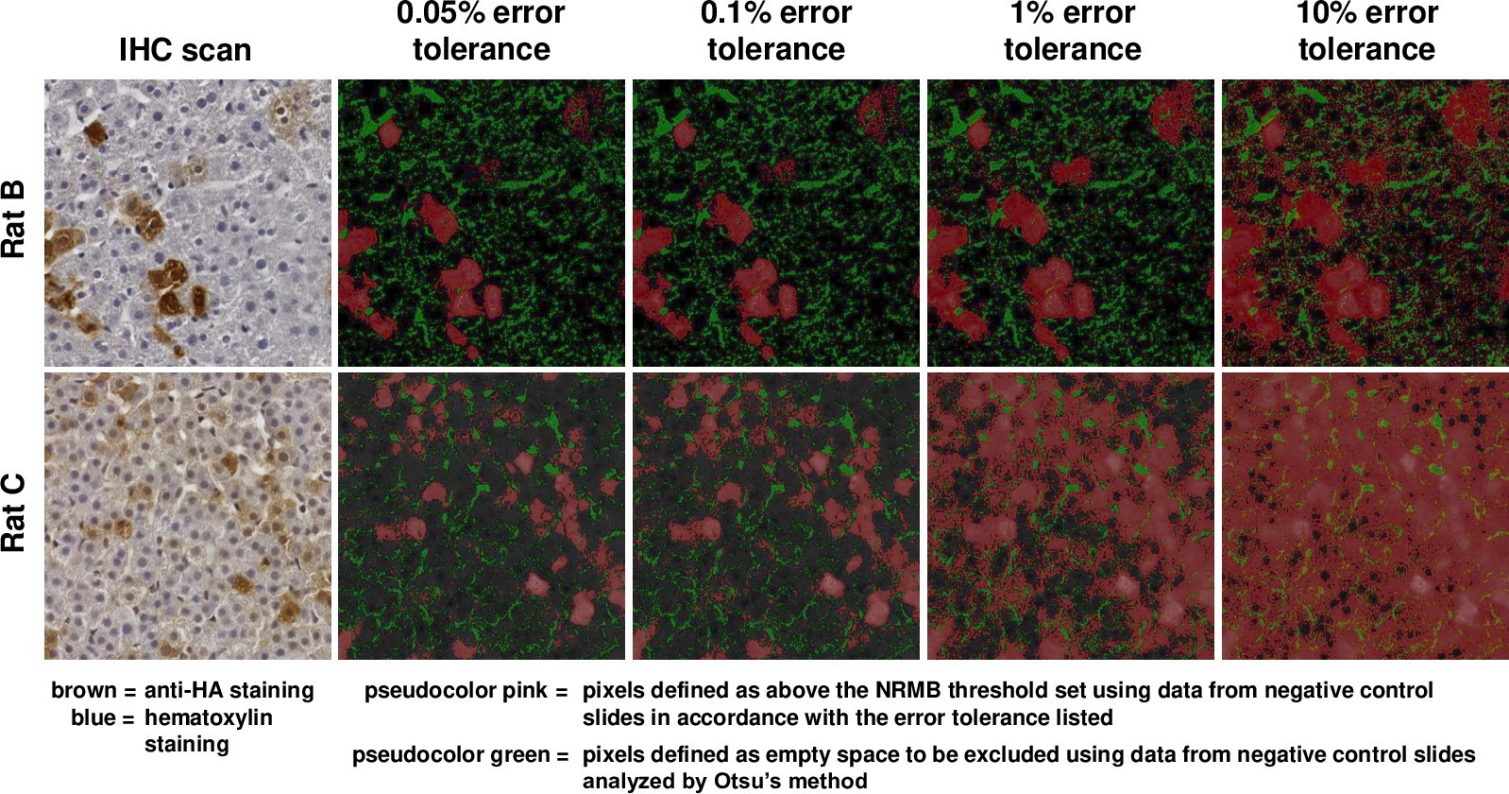
Visual representation of the impact of changing error tolerance limits on the definition of DAB-stained versus unstained tissue pixels in 2 test slides. Panels on the left are true color. All other panels present the same images in pseudocolor, with green representing pixels deemed empty space, pink representing pixels deemed DAB-stained tissue, and dark gray representing pixels deemed unstained tissue.

Fig 2 illustrates the relationship between visible DAB staining in a section of test slide and the pixels designated as “stain positive” (pink pseudocolor) given different error tolerance limits. As expected, the higher the error tolerance, the more pixels are counted as stained (Fig 2 and Table 2). The green color in each panel represents pixels designated as “empty space;” these designations were set by Otsu’s method using the control slides and do not change with increasing user-defined error tolerance.

**Table 2:** Impact of error tolerance setting on fraction of pixels that exceed the resulting NRMB threshold for DAB staining.

% Error Tolerance Limit	NRMB threshold	Fraction of pixels that exceed the NRMB threshold in example slides with lower and higher staining levels: Rat B (low signal) Rat C (high signal) (20 regions) (20 regions)	
0.05%	0.334818	0.037580	0.236033
0.1%	0.265088	0.040969	0.336549
1%	0.115669	0.119639	0.798243
10%	0.035978	0.338658	0.964708

### Full image quantitation versus random sampling

The fraction of pixels stained in a tissue sample can be determined either by calculating the NRMB for every tissue pixel on the slide, or by sampling the tissue pixels using small boxes of defined size scattered randomly and automatically across the slide. For sampling, regions are selected by subdividing the entire slide into a grid and rejection sampling regions at random without replacement until the user-specified number of regions has been processed. A candidate region is rejected automatically if more than a user-specified fraction of its pixels is classified by Otsu’s method as empty space (default = 0.5).

**Fig 3:**
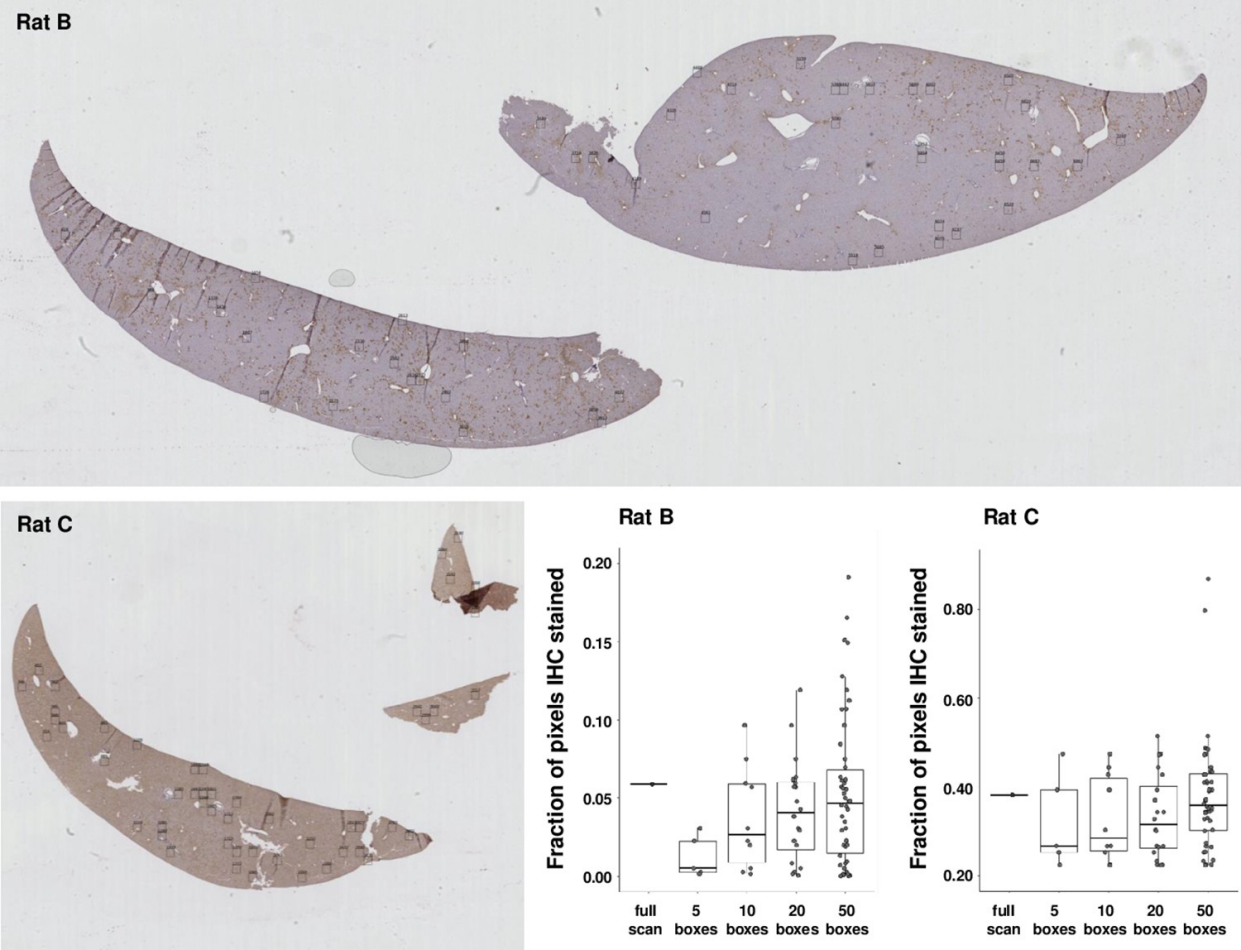
Fraction of DAB-stained tissue pixels in each of 2 test slides quantified by whole slide analysis versus random sampling with 5, 10, 20, or 50 boxes per slide. With an increasing number of boxes, the median fraction of DAB-stained pixels approaches the fraction calculated by whole slide analysis. Random sampling dramatically cuts processing time and reveals the level of heterogeneity in tissue staining.

Fig 3 presents the results of full scan versus random sampling analyses of 2 slides, one showing a relatively high fraction of stained pixels (from Rat C), the other a relatively low fraction of stained pixels (from Rat B). For each of these slides, a full scan yielded the highest fraction of pixels deemed stained, but random sampling of 50 boxes yielded a median value similar to the full scan, at a fraction of the run time (seconds versus minutes, see Table 1). Further, as explained below, and illustrated in S1 Fig, the full-scan fraction of stained tissue pixels may be an over-estimate if the slide contains artifactually hyper-stained regions, such as wrinkles or folds in the tissue.

### Technical reproducibility of sampling

**Fig 4:**
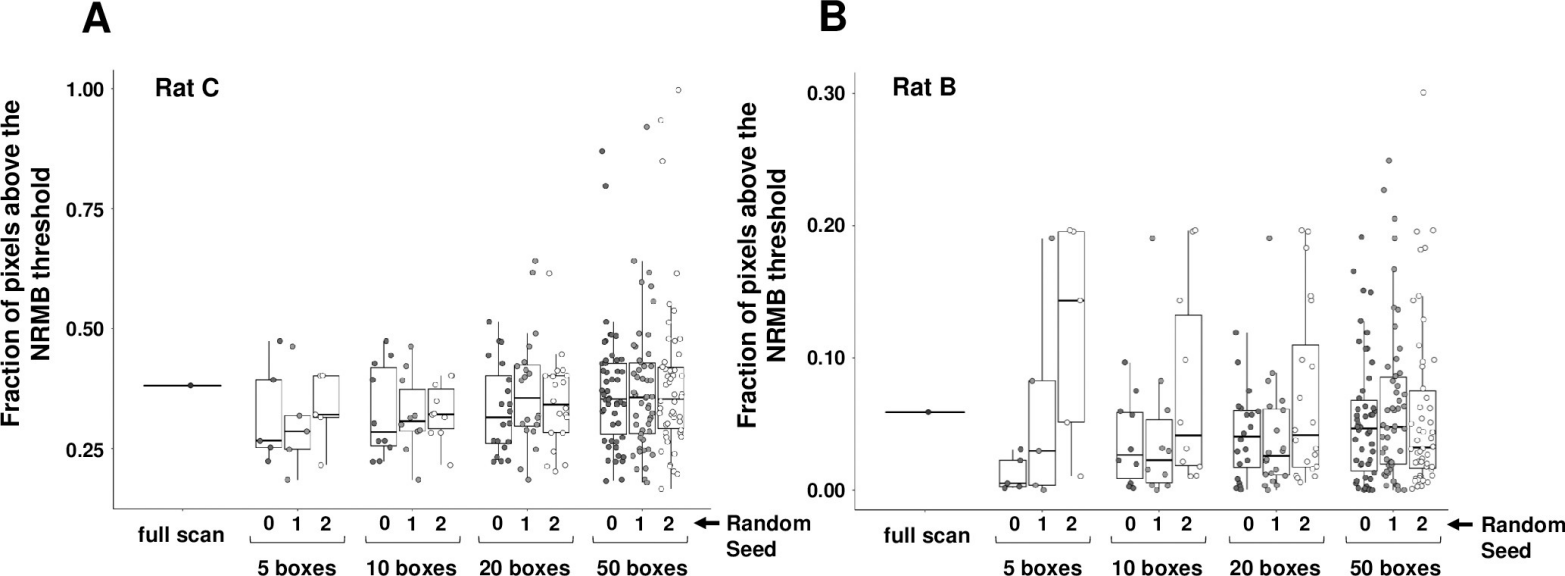
Reproducibility of the fraction of pixels deemed DAB stained improves with an increasing number of sampling boxes. Random sampling with the indicated number of boxes per slide was repeated using each of 3 different randomization seeds to test reproducibility in each of 2 different slides.

To test the technical reproducibility of automatic sampling in our system using 5, 10, 20, or 50 boxes per slide, we repeated each sampling exercise 3 times, each time using a different seed to drive the randomization process so the sampling boxes would be independent. As illustrated in Fig 4, individual boxes showed sometimes very different fractions of stained pixels, but as the number of boxes increased the median proved increasingly reproducible.

### Option to exclude specific boxes

**Fig 5:**
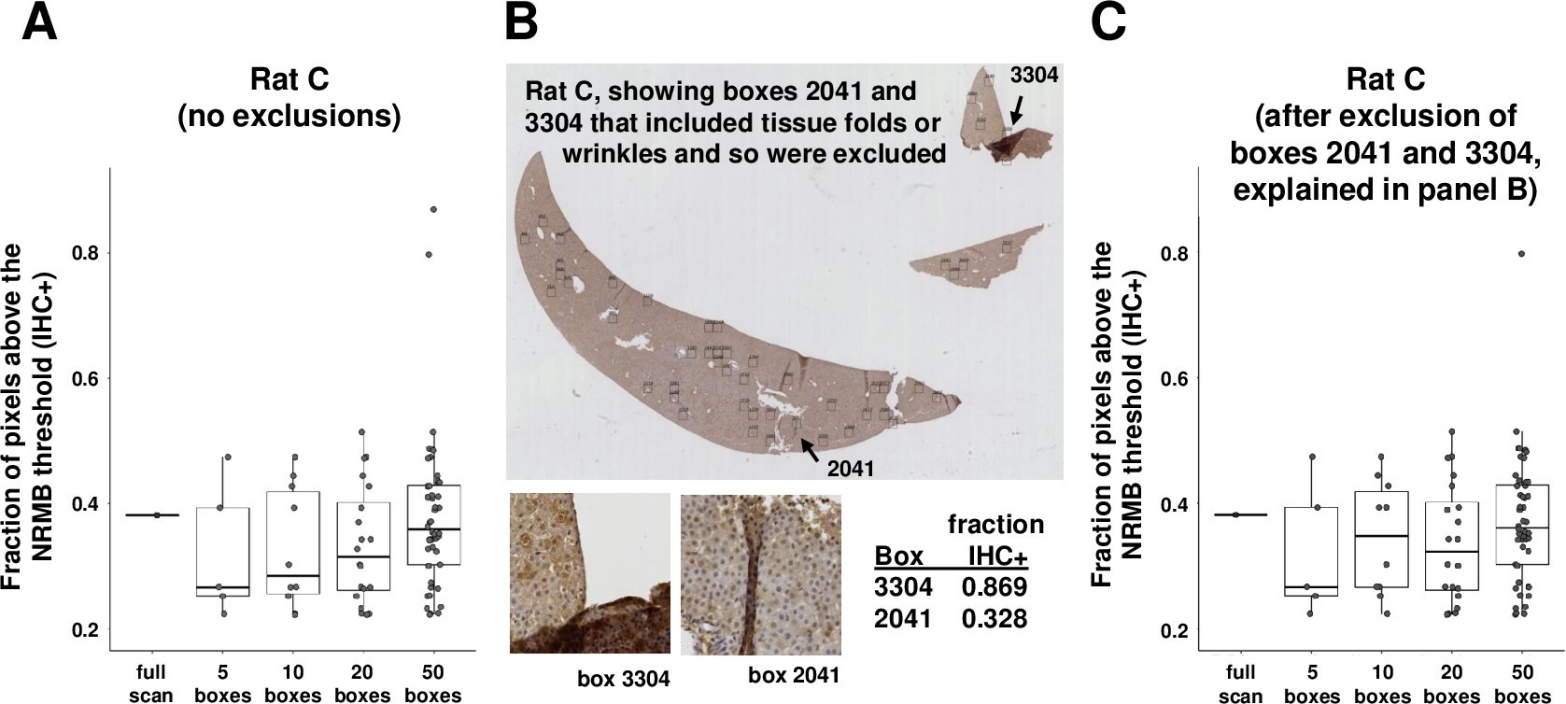
Excluding problem areas of slides. Sampling boxes allow the user to exclude and replace regions of slides deemed compromised, for example, by wrinkles or folds in the tissue. In this slide, there were only minimal problem areas visible (panel B) so the fraction of DAB-stained pixels estimated using random sampling boxes without exclusions (panel A) is similar to that estimated with exclusions (panel C). The impact could be larger for a more compromised slide.

Finally, we tested the possibility of manually excluding specific sampling boxes from analysis if they fell on regions of the slide deemed compromised, for example by visible wrinkles or folds in the tissue (e.g., Fig 5, boxes 2041 or 3304). To exclude specific boxes from scoring, the corresponding ID numbers are entered into a csv file that is created automatically when a test slide is processed using a given randomization seed. If the total number of sampling boxes used for a given slide is high (e.g., 50) and the imperfections are few, excluding problem boxes may have little impact on the overall median DAB-stained fraction (Fig 5), but in instances where imperfections are many, this may be a useful feature.

S1 Fig provides further examples of analyses with and without box exclusion for 2 additional slides, one of which includes many visible imperfections. As expected, whole slide analysis, which contains all folds and wrinkles, yields the highest score of stained pixels for both slides illustrated; analysis of 50 sampling boxes scattered randomly across the tissue yields a slightly lower score for both slides; and finally, analysis of 50 sampling boxes after exclusion and replacement of original boxes that included folds or wrinkles yields a score that is lower still in both slides.

## Discussion

Here we describe DAB-quant, a novel open-source system designed to facilitate the quantitation of DAB staining in large numbers of tissue slides with a minimum of user effort. To be clear, all of the data presented here were generated using scans with a.ndpi extension, generated using a Hamamatsu scanner. However, DAB-quant allows the user to define other file extensions and, at least in theory, the system should work with any file format compatible with OpenSlide. We were able to test scans with an extension of.qptiff, generated using an Akoya Vectra Polaris scanner, and they also worked. Scans generated using different instruments may demonstrate differences in color balance, or other characteristics, and so may yield slightly different results.

Our system applies a set of negative control slides, and a user defined error tolerance, to set thresholds for distinguishing empty space from stained and unstained tissue pixels. Of course, variation in staining intensity between slides–even negative controls–may reflect technical as well as biological factors. That DAB-quant allows the user to score many negative control slides in composite to set thresholds is a strength of our system. Ideally, these negative control slides would represent the same staining batches used to generate the test slides to be scored. Further, the ability to score either full slides, or randomly sampled areas within slides, enables more efficient processing as well as insight into the potential heterogeneity of staining across the tissue surface. Finally, the ability to exclude specific areas from scoring permits the analysis of slides with multiple visible imperfections that might otherwise confound analysis. One example presented here (Rat D in S1 Fig) illustrates this point.

Our scoring paradigm applied here, calculating normalized red minus blue intensity per tissue pixel, was devised to quantify DAB staining (brown color) in tissues also treated with hematoxylin (blue color). Other scoring paradigms also work well for this type of application [10–12], though for most, analysis requires more user input per slide which can become challenging when very large numbers of slides need to be scored. DAB-quant allows the user simply to deposit all of their test slides in one folder, and all of their negative control slides in a separate folder. Scoring 100 slides therefore takes essentially the same level of user input as scoring 10 slides.

Because our code is open source, the scoring strategy we devised can be modified by users to work for other color schemes by changing how the red, green, and blue channel intensities of each pixel are handled. For example, normalized green minus blue could be used to detect green staining. For staining that is not well aligned with the red, green, and blue color channels, it should be possible to project the relevant colors onto a color vector chosen by principle or independent component analysis, or by color deconvolution [13] as in QuPath [5]. Of note, our scoring system allows both ordinal (histogram) and binary (fraction stained) classification of pixels. For different applications, or in the context of specific challenges such as low signal-to-noise ratio, one or the other of these options might be preferable.

The 4 test slides analyzed here were selected because they represent different levels and distribution patterns of DAB staining or different degrees of slide imperfections (wrinkles and folds). All of these slides present liver tissue from GALT-null rats [14] that were administered scAAV9-hGALT virus shortly after birth and then euthanized for tissue collection at either 14 (Rats C, D, E) or 30 (Rat B) days post-treatment. The 6 negative control slides include liver tissue from GALT-null rats administered phosphate buffered saline (PBS) rather than virus shortly after birth, and harvested in parallel with their treated counterparts. As described elsewhere [7], we saw a higher fraction of DAB-stained cells with a broader distribution of staining intensities at 14 days, and fewer stained cells with a tighter distribution of staining intensities at 30 days. The histograms presented in Fig 1 illustrate these patterns.

Our scoring system has many strengths, perhaps the greatest being ease of use with large numbers of slides, and ability to exclude problem areas without also having direct user-selection of regions to be quantified. Our system also has clear limitations. For example, color intensity is quantified by pixel, not cell, so the result is only a close proxy for staining per cell if the population of cells is reasonably uniform in area. Similarly, as written, DAB-quant was not designed for detailed analyses of segmentation and will not distinguish differential staining among cell types in the same slide, or differential subcellular location of staining within cells. Of course, given double-label staining it might be possible to alter the code to quantify pixels based on specified combinations of colors rather than simply normalized single colors, as was done here.

## Supporting information

S1 File Text and GitHub link for DAB-quant published on protocols.io at DOI: dx.doi.org/10.17504/protocols.io.dm6gpb578lzp/v1. (DOCX)

S1 Fig Images and fraction of stained pixels scored in 2 additional test slides before and after exclusion of sampling boxes that included tissue wrinkles or folds. (TIF)
