# Supplementary material for: DAB-quant: An open-source digital system for quantifying immunohistochemical staining with 3,3′-diaminobenzidine (DAB)
Source: PLoS One. 2022 Jul 20;17(7):e0271593. doi: 10.1371/journal.pone.0271593 (PMC9299305; doi:10.1371/journal.pone.0271593)
Supplement: S1 File — (DOCX) [file pone.0271593.s001.docx]

Text available on protocols.io at [dx.doi.org/10.17504/protocols.io.dm6gpb578lzp/v1](https://dx.doi.org/10.17504/protocols.io.dm6gpb578lzp/v1)

Here we provide DAB-quant, a new system that facilitates quantitation of large numbers of scanned tissue slides stained via immunohistochemistry with 3,3′-Diaminobenzidine (DAB). The python code, instructions, license, and a link to example scans for analysis are all available at: https://github.com/sarafridov/DAB-quant
