# Supplementary figures and images for: DAB-quant: An open-source digital system for quantifying immunohistochemical staining with 3,3′-diaminobenzidine (DAB)
Source: PLoS One. 2022 Jul 20;17(7):e0271593. doi: 10.1371/journal.pone.0271593 (PMC9299305; doi:10.1371/journal.pone.0271593)

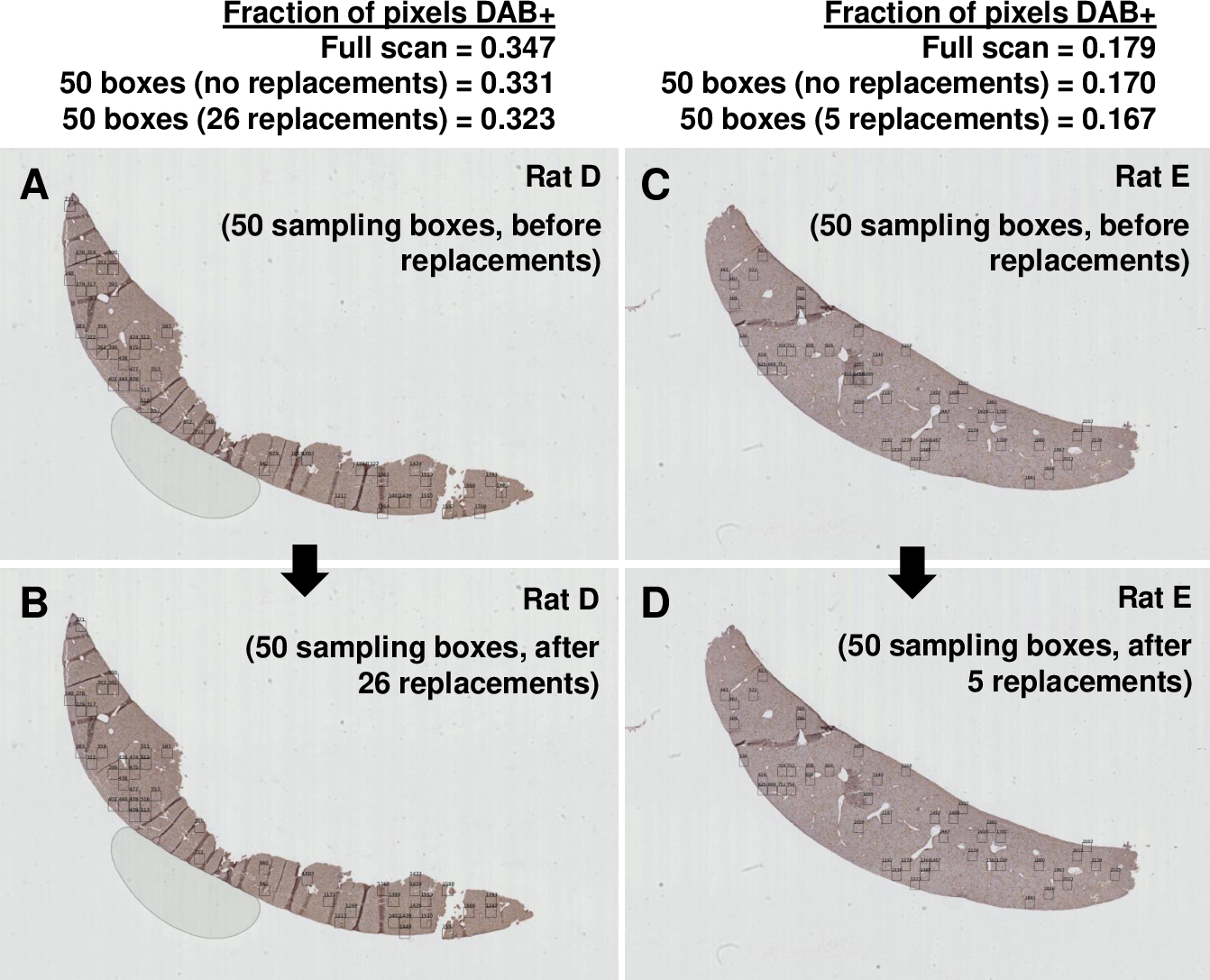

Supplement: S1 Fig — (TIF) [file pone.0271593.s002.tif]
